# Supplementary material for: Reduced Risk of Oat Grain Contamination with Fusarium langsethiae and HT-2 and T-2 Toxins with Increasing Tillage Intensity
Source: Pathogens. 2022 Nov 3;11(11):1288. doi: 10.3390/pathogens11111288 (PMC9698665; doi:10.3390/pathogens11111288)
Supplement: Supplementary file 1 [file pathogens-11-01288-s001.zip › pathogens-1957902-supplementary.pdf]

Table S1: The mean concentration of *Fusarium* DNA and mycotoxins in oat grain harvested year 2011 and 2012 from field plots receiving various tillage and straw removal treatments over a three-year period (2010-2012).

Field[1]	Treatm.[2]	Fl DNA[3,4] (pg/µg)	(STD)[5]	Fg DNA[3,4] (pg/µg)	(STD)	Fa DNA[3,4] (pg/µg)	(STD)	DON[6,4] (µg/kg)	(STD)	HT2+T2[6,4] (µg/kg)	(STD)	ENNB (µg/kg)	(STD)	ENNB1 (µg/kg)	(STD)	BEA (µg/kg)	(STD)	NIV (µg/kg)	(STD)
Solør	DAP	1707	(743)	2518	(570)	6928	(1971)	573	(122)	165	(122)	NA[7]		NA		NA		NA	
2011	SSP	1360	(751)	2109	(1032)	5436	(2641)	342	(154)	161	(144)	NA		NA		NA		NA	
	SAH	3030	(1746)	1419	(1131)	6556	(3207)	233	(167)	228	(122)	NA		NA		NA		NA	
	SSH	2560	(1893)	691	(460)	10348	(6636)	189	(149)	205	(149)	NA		NA		NA		NA	
Solør	DAP	380	(129)	284	(37)	659	(322)	854	(447)	99	(40)	131	(85)	15	(9)	16	(14)	210	(89)
2012	SSP	598	(469)	520	(435)	250	(124)	1012	(876)	170	(84)	48	(45)	6	(7)	20	(22)	196	(75)
	SAH	1702	(1498)	135	(38)	891	(94)	610	(233)	340	(167)	97	(51)	10	(4)	17	(14)	157	(53)
	SSH	2211	(429)	53	(36)	615	(446)	358	(193)	476	(165)	61	(58)	6	(7)	21	(14)	138	(147)
Østfold	DAP	1954	(288)	3375	(1882)	3572	(738)	2102	(2131)	34	(11)	NA[7]		NA		NA		NA	
2011	SSP	2845	(504)	4535	(648)	2243	(781)	1469	(1368)	98	(34)	NA		NA		NA		NA	
	SAH	2884	(857)	3471	(3538)	1543	(562)	2001	(3647)	152	(23)	NA		NA		NA		NA	
	SSH	2710	(463)	1519	(2021)	1629	(1158)	247	(41)	196	(34)	NA		NA		NA		NA	
Østfold	DAP	919	(473)	255	(145)	141	(49)	750	(777)	484	(180)	26	(16)	9	(5)	11	(8)	197	(79)
2012	SSP	833	(244)	302	(228)	341	(156)	338	(100)	302	(102)	18	(21)	4	(4)	8	(8)	187	(42)
	SAH	2375	(1718)	89	(57)	402	(502)	410	(267)	780	(512)	8	(17)	2	(3)	4	(6)	210	(115)
	SSH	1767	(1065)	179	(126)	328	(338)	453	(165)	564	(313)	140	(229)	4	(5)	7	(9)	248	(93)

1: The grain was harvested from non-inoculated experimental fields of oats at two locations in southeast Norway (Solør and Østfold) in 2011 and 2012.
2: The following tillage methods were included: DAP = deep autumn ploughing; SSP = shallow spring ploughing; SAH = shallow autumn harrowing; SSH = shallow spring harrowing.
3: DNA concentration of *Fusarium avenaceum* (Fa), *Fusarium graminearum* (Fg), and *Fusarium langsethiae* (Fl) in grain harvested from plots receiving the different tillage treatments. The DNA concentration is presented as pg DNA of the respective *Fusarium* species per µg plant DNA.
4: Each value is the average result from four plots receiving similar tillage treatment within a field. In two of these plots the straw was removed whereas in the other two plots the straw was chopped and retained in the field.
5: STD = standard deviation.
6: Mycotoxin concentration (µg/kg) in grain harvested from plots receiving the different tillage treatments. DON = deoxynivalenol, HT2+T2 = The sum of HT-2 and T-2 toxins, ENNB = Enniatin B, ENNB1 = Enniatin B1, BEA = beauvericin, NIV = nivalenol.
7: NA = Not analysed.
